# Pushout Bond Strength of Root Fillings after Irrigation of Root Canals Utilizing Sodium Hypochlorite, Chlorhexidine, and Homeopathic Mother Tincture (*Arnica Montana*)

**DOI:** 10.3390/clinpract13010028

**Published:** 2023-02-17

**Authors:** Unmesh Khanvilkar, Hitesh Patil, Siddhesh Bandekar, Shirin Kshirsagar, Ajinkya M. Pawar, Dian Agustin Wahjuningrum, Francesco Pagnoni, Rodolfo Reda, Alessio Zanza, Luca Testarelli

**Affiliations:** 1 Department of Conservative Dentistry and Endodontics, Yogita Dental College and Hospital, Khed, Ratnagiri 415709, Maharashtra, India; 2 Department of Conservative Dentistry and Enododntics, Nair Hospital Dental College, Mumbai 400008, Maharashtra, India; 3 Department of Conservative Dentistry, Faculty of Dental Medicine, Universitas Airlangga, Surabaya City 60132, East Java, Indonesia; 4 Department of Oral and Maxillofacial Sciences, Sapienza University of Rome, 00161 Rome, Italy; 5 Department of Prosthodontics and Implantology, Saveetha Dental College and Hospitals, Saveetha Institute of Medical and Technical Sciences, Chennai 600077, Tamil Nadu, India

**Keywords:** AH Plus sealer, *Arnica montana*, chlorhexidine, pushout bond strength, radicular dentin, sodium hypochlorite

## Abstract

The pushout bond strength of root fillings at radicular dentin was investigated employing NaOCl, CHX, and homoeopathic mother tincture (*Arnica montana*) as an irrigant. Sixty human permanent single-rooted extracted teeth were decoronated. The root canals were instrumented using Pro taper universal rotary system (Dentsply Tulsa Dental; Tulsa, Oklahoma) and were prepared up to F3 apical size. The roots were then randomly divided into three groups according to irrigation solution (*n* = 20) according to the final irrigation regimen: Group I: 3 mL 5.25% NaOCl followed by 3 mL Saline (control); Group II: 3 mL *Arnica montana* (10%, *w*/*v*) followed by 3 mL Saline; Group III: 3 mL CHX followed by 3 mL Saline. The canals were dried using paper points. The canals were coated with AH Plus sealer (Dentsply DeTey, Konstaz, Germany) with the aid of a Lentulo spiral (Dentsply DeTey, Konstaz, Germany) and obturated with #F3 gutta-percha. Each root was then horizontally sliced into three slices, labelled coronal, middle, and apical, each measuring 2 mm thick. Furthermore, at a crosshead speed of 2 mm/min, the test was carried out using the universal testing apparatus. The 5.25% NaOCl significantly decreased the bond strength of AH Plus to dentin. Both CHX and *Arnica montana* were capable of reversing the compromised pushout of AH Plus to NaOCl-treated dentin. After using NaOCl as an irrigant, the danger of diminished binding capacity of AH Plus to root canal walls arises. Final irrigation with *Arnica montana* and CHX reduces this risk.

## 1. Introduction

A thorough chemomechanical preparation of the root canal system, the removal of pathogenic organisms, and a three-dimensional filling of the canal space with an inert root filling material are necessary for a successful root canal procedure. This helps to dissuade microorganisms from entering the canal from the oral cavity and spreading to the periapical tissue [1]. The dentinal tubules and canal dentinal surfaces are often covered by an amorphous uneven smear layer after biomechanical preparation. This smear layer must be removed since it is home to pathogens and necrotic tissues. It serves as a barrier and prevents irrigants from penetrating into dentinal tubules. This weakens the binding strength of root canal sealers, leading to microleakage and eventual failure of root canal treatment [1,2].

The root canal dentin cannot be adhered to by gutta-percha alone. In order to adhere it to the root canal dentin, sealers are employed [1]. For endodontic application, a variety of sealers are available, ranging from early ZOE-based sealers to epoxy resin-based sealers. Due to its favorable physical features, decreased solubility, appropriate biological performance, superior sealing ability, and greater micro-retention to root dentin, epoxy resin-based sealers like AH Plus are gradually surpassing other sealers [1]. The most commonly implemented root canal irrigant is sodium hypochlorite (NaOCl), which has tissue-dissolving and antimicrobial characteristics. NaOCl has been linked to a number of undesirable effects, including an unpleasant taste and odor, toxicity, potential paresthesia of the mandibular nerve, allergies, dentin deterioration through collagen breakdown, and an increase in coronal microleakage of adhesive restorations. When used as a final irrigant, it has been demonstrated that NaOCl inhibits the polymerization of AH Plus sealers [2]. However, the employment of an additional chemical agent with such qualities has piqued curiosity due to its inability to dissolve inorganic substances. Therefore, to remove this layer, certain chemical agents are utilized, such as EDTA solutions at concentrations ranging from 15 to 17%, citric acid (5–50%), and phosphoric acid (5–37%) [2]. Because it has strong antimicrobial properties, chlorhexidine (CHX) is used. However, CHX lacks tissue-dissolving properties. As reported, by using it as the final irrigant, resin-based sealants may be made to adhere better to dentine surfaces and coronal microleakage can be avoided [3,4]. Due to their strong antimicrobial properties, biocompatibility, and antioxidant and anti-inflammatory properties, herbal and natural products have been used in dentistry and medicine for thousands of years and have only grown in popularity in recent years [5].

In response to the negative consequences of synthetic medications, experts are looking for natural substitutes. Herbal remedies used in endodontics provide a number of benefits, including affordability, use, and better storability. Herbs used as medicine are thought to have bioactive components. It is possible to cure infectious disorders with some organic plant extracts. They may be utilized as endodontic irrigants and intracanal medicines since they are biocompatible and lessen the negative effects of synthetic antimicrobials. The majority of herbal irrigants are also nontoxic and safe for host tissues [5,6]. 

*Arnica montana* is a natural substance with several applications in phytotherapy. Since ancient times, *Arnica montana* has been utilized as an herbal remedy. Preparations containing arnica have mostly been proven to alleviate tenderness, inflammation, and discoloration brought on by sprains and bruises. Long used topically (applied externally), the herb’s dried flower heads and roots are used to make tinctures (included in gels), infusions, and a broad array of ointments. Products made with *Arnica montana* show the potential to destroy intracanal micro-organisms [6]. Because of research demonstrating that Asteraceae-containing therapies were often used as part of German essential care and that their usage was unrelated to toxic reactions, *Arnica montana* is regarded as safe for human administration [7]. Arnica has been used to effectively ease post-tooth extraction discomfort. Arnica works locally on the muscles, joints, and blood vessels to reduce inflammation, pain, and pain in aggregate [7]. Its value as an anti-inflammatory agent has been established. Additionally, *Arnica montana* has antibacterial efficacy in a study reported by Loo et al. [8]. The presence of thymol derivatives has been linked to bactericidal properties [7]. There has lately been a gradual transition from the previously employed synthesized chemical components towards natural herbal compounds. As a result, we may simply substitute herbal irrigation material for sodium hypochlorite. As canal irrigation with diverse chemical solutions results in structural changes, as seen by the decline in dentine microhardness [9], *Arnica montana* can be considered an influential irrigation solution.

The success of endodontic treatment is compromised due to leakage of irritants to the periapical region. Adhesive property of an endodontic sealer is important to minimize the detachment of material from the dentin and to prevent leakage, which promotes successful endodontic therapy. Bond strength tests are done in endodontics to test the adhesiveness of the endodontic material to the tooth surface. Increased adhesive properties of the material may provide greater strength of restored teeth [9].

Being applied to root canal dentine, pushout bond strength often indicates the degree of resistance to the dislodgment of a filling substance. A tensile load is oriented vertically to the long axis of the root until the filling is displaced [10] in order to achieve pushout bond strength. According to Üregan et al. [11], pushout bond strength provided a more accurate estimate of bond strength than traditional shear testing. Even though pushout bond strength test might be less accurate in reflecting the clinical situation of the sealers, it is presently the finest adhesion test available. In contrast to other approaches, which may include numerous core materials and preparation methods for root dentine, this test is simple to administer, evaluate, and record [12].

To the best of the authors’ awareness, there hasn’t been any research conducted to date to assess the pushout bond strength of AH Plus/gutta-percha by employing homoeopathic medications like *Arnica montana* as a root canal irrigant. As a result, the goal of this study is to determine the pushout bond strength of the resin sealer at the radicular dentin when sodium hypochlorite is used in combination with a homoeopathic mother tincture as an irrigant.

## 2. Materials and Methods

The current study was approved by the Institutional Ethics Committee of Yogita Dental College and Hospital (YDCH/2017/1925/2022; date of approval 12 October 2022) and conducted in the Department of Conservative Dentistry and Endodontics of Yogita Dental College and Hospital, Khed, Maharashtra, India. Sixty single-rooted mandibular premolar teeth with fully developed apices that were excised for orthodontic purposes were employed in the current investigation. X-rays were used to select teeth exhibiting only one canal with no cracks or fractures found under an operating microscope (×10) and displaying no dilaceration or calcification and were immediately stored in a 0.9% thymol solution until use. To obtain a standardized length of 15 mm, the teeth were decoronated at a cementoenamel junction using a low-speed handpiece (NSK, Kanuma, Japan) and diamond disc underwater cooling. By subtracting 1 mm from the root canal length, working lengths were established. This was followed by standardized biomechanical preparation of the canals using ProTaper Universal Rotary Instruments (Dentsply Sirona) up to F3 apical size. The specimens were then randomly divided into three groups (*n* = 20) according to the final irrigation regimen, as follows:

- Group I: 3 mL 5.25% NaOCl (Prime Dental Pvt. Ltd., Mumbai, India) followed by 3 mL saline (control).
- Group II: 3 mL *Arnica montana* followed by 3 mL saline.
- Group III: 3 mL 2% CHX (Prevest DenPro Ltd., Jammu, India) followed by 3 mL saline.

### 2.1. Root Canal Irrigation

A 30-gauge side vent needle and syringe were used to irrigate all the test group irrigating solutions. For each experimental group, the samples were irrigated with 3 mL of the experimental solutions for 2 min followed by a flush of 3 mL saline. 

### 2.2. Preparation of Arnica Montana Solution

To synthesize ethyl extract for root canal irrigation, the arnica flowerheads were used to obtain arnica extract. The gathered plant samples were crushed to a fine aggregate in an electric grinder and kept in a tightly covered container after drying in the open air for 15 days under shelter. 100 g of each powdered plant material was soaked in 900 mL of 70% ethanol to create homoeopathic mother tinctures. The soaked plant material from each plant was filtered after 15 days, and mother tincture was bottled and employed as irrigation [13].

**Figure 1:**
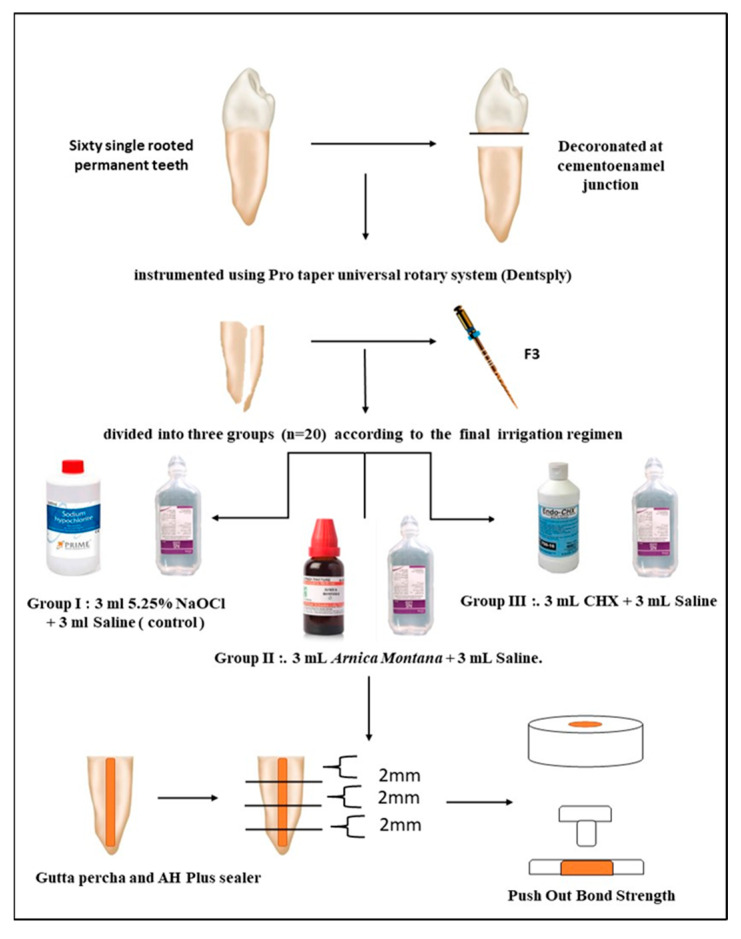
Flow chart of the study. (NaOCl: Sodium hypochlorite; CHX: Chlorhexidine; mL: milliliters; and mm: millimeters).

Normal saline was used after each experimental solution. It serves to cleanse the root canal of the preceding irrigation solution and has no antibacterial properties [4]. Following irrigation protocol with the experimental solutions, the canals were irrigated using 3 mL 17% aqueous EDTA (DentWash, Prime Dental Products Pvt. Ltd., Mumbai, India) enhancing smear layer removal [4]. Following this, all groups were dried with absorbent paper point. The root canals were obturated using gutta-percha and AH Plus sealer (Dentsply DeTrey GmbH, Germany). According to the manufacturer’s instructions, the sealer was mixed. Obturation was achieved by single-cone technique with gutta-percha. Using a low-speed saw (Buehler, Lake Bluff, NY, USA) with a diamond disc (Swiss NF Metals, Markham, ON, Canada), each sample was horizontally sectioned into 1 mm-thick slices at the coronal third, middle third, and apical third of the root. Three different plungers of 1 mm, 0.8 mm, and 0.3 mm diameter were used for coronal, middle, and apical sections, respectively. To avoid any constriction interference, the load was always applied in an apical–coronal direction that may have been caused by the root canal taper during pushout testing. The test was performed using the universal testing machine at a crosshead speed of 2 mm/min, and the load required to dislodge the root fillings was recorded for each sample (Figure 1).

After evaluating pushout bond strength using a stereomicroscope (Olympus SZ61, Olympus Optical Co., Tokyo, Japan), the failure modes of debonded specimens were analyzed. Using the following criteria, the failure modes were classified according to Fowler et al. [14]:

1. Adhesive failure between sealer and dentin (when the dentin was more than 75% free of root-filling material).
2. Cohesive failure within sealer (when the percentage dropped below 25%).
3. Mixed failure (when the percentage of exposed dentin was between 25% and 75%).

### 2.3. Statistical Analysis

Data entry was performed using Microsoft Excel 2010. Descriptive statistics were expressed as mean ± standard deviation (SD) for each group for pushout bond strength. Three groups (Group A1 vs. Group A2 vs. Group A3) and (Coronal vs. Middle vs. Apical) were compared for pushout bond strength by analysis of variance (ANOVA) followed by Tukey’s post hoc test for pairwise comparison. Simple/multiple bar charts were used for graphical representation. The statistical significance level was set at *p* < 0.05. The Statistical Package for Social Sciences (SPSS, Chicago, IL, USA) version 19 software was used for analysis.

## 3. Results

The pushout bond strength values of the three groups are presented in Table 1. It was observed that in all groups, the bond strength in the coronal third was higher than the apical third (*p* < 0.001). In canals irrigated with sodium hypochlorite, the bond strength values of root fillings that were performed with AH Plus sealer were 1.7630 (±0.44502),1.8730 (±0.49376), 1.7850 (±0.39697). The MPa differed statistically in the coronal, mid-root and apical parts (*p* < 0.001). In canals irrigated with *Arnica montana*, the bond strength values of root fillings that were performed with AH Plus sealer were 5.2480 (±0.65287, 4.4630 (±0.85853), 4.3110 (±0.91307) MPa in the coronal, mid-root and apical parts, respectively (*p* < 0.001). In canals irrigated with chlorhexidine, the bond strength values of root fillings that were performed with AH Plus sealer were 4.9960 (±0.67145), 4.6010 (±1.45383), 4.2390 (±0.82174) MPa in the coronal, mid-root, and apical parts, respectively (*p* < 0.001).

### Intragroup Comparisons

In intragroup comparison for mean pushout bond strength between three levels (coronal, middle, and apical) among Group 1 (sodium hypo + saline), there was statistically insignificant difference, with *p* = 0.845. 

In intragroup comparison for mean pushout bond strength between three levels (coronal, middle, and apical) among Group 2 (*Arnica montana*), there was statistically significant difference, with *p* = 0.035. There was statistically significant difference for mean pushout bond strength between the coronal level and apical level among Group 2, with *p* = 0.041 (Table 1). 

In intragroup comparison for mean pushout bond strength between three levels (coronal, middle, and apical) among Group 3 (CHX), there was statistically insignificant difference, with *p* = 0.282 (Table 1).

The mean with standard deviation and the statistical analysis by application of one-way ANOVA are presented in Table 1. The intragroup comparisons are presented in Table 2. The pushout bond strength in the apical section, when compared to the coronal section, was significantly higher in Group 2.

The failure analysis showed that mixed failure mode was predominant among the experimental groups and the adhesive failure mode was predominant in the control group (Table 3).

## 4. Discussion

The comprehensive sealing of the root canal system is necessary for endodontic therapy to be successful [15]. Obturation is the process of filling the complete root canal system, which requires the use of materials with physicochemical and biological qualities to seal the canal both apically and coronally [16]. Root canal fillings’ primary purpose is to prevent leakage into the root canal system from the oral cavity and peri-radicular tissue [16]. While sealer is necessary to aggregate the filling material, maintain compact mass without voids, bind it to the canal wall, and give single unit configuration, gutta-percha is a biocompatible substance that is used to fill radicular space [17]. The most popular technique for evaluating the efficacy of the binding capability between endodontic root-filling materials and radicular dentin is bond strength testing [18,19]. In order to determine their impact on the pushout bond strength of the AH Plus sealer with root canal obturation, several irrigation schedules were applied in the current investigation.

From coronal to apical direction, the bond strength decreased in the current research. Our findings, when compared to those of several studies, show that root canal sealants often have poorer binding capacity in the coronal to apical direction [20,21,22]. Smear layer forms during biomechanical preparations. Smear layer serves as a microbial reservoir and can prevent sealer tags from extending into dentinal tubules, which reduces micromechanical adhesion. Smear layer should be eliminated as an outcome [23]. The highest adhesive force readings are linked to the smear layer removal [21].

The organic debris in the canal is removed during instrumentation by the application of NaOCl. It eventually results in the collagen of the root dentin degrading as well. NaOCl degrades into chloramines and protein-derived radical intermediates when it comes into contact with organic matter in the root canal [24]. As reported by Daumer et al., these breakdown products have the potential to negatively impact the pyridinoline crosslinks seen in type I collagen. Epoxy resin sealer has been proven to chemically link to the amino groups of dentin; hence, collagen is necessary for its adherence. When NaOCl is used as a last rinse prior to the application of AH Plus in the canal, this adhesion process is impeded. Another reasonable explanation is that the oxygen bubbles that were created after using NaOCl prohibit the epoxy resin sealer from entering the tiny orifices of dentinal tubules [25].

The strength of the dentin bond following sodium hypochlorite treatment has been linked to the deproteinizing effect of sodium hypochlorite in the NaOCl group. The uncovered dentinal collagen may be broken down and removed with NaOCl, creating a new surface of mineralized dentin on which the adhesive resin can be put. As a result, the resin-reinforced collagen layer known as the “hybrid layer” is not necessary for the adhesive resin and dentin to adhere directly to one another. As a result, the adhesive resin will penetrate the mineralized matrix and fill the submicron-sized porosities. This develops a layer of mineralized matrix that is penetrated with resin [26].

*Arnica montana* irrigation greatly increased the bond strength of AH Plus to dentin in this investigation. Significant antioxidant activity demonstrated by *Arnica montana* has been evaluated using the phosphomolybdate technique and the 2,20-diphenyl-1-picrylhydrazyl (DPPH) free radical scavenging method [27]. According to the phosphomolybdate technique, *Arnica montana* exhibits 71.52% DPPH scavenging capacity and 63.68% total antioxidant activity at a dosage of 5 mg/mL. Flavonoids and phenolic chemicals are mostly responsible for this [27]. Due to its antioxidant and anti-inflammatory qualities, which counteract the harmful effects of free radicals, it displayed the greatest binding strength to resin sealer. These antioxidant qualities enable the adhesive’s free-radical polymerization to continue without premature termination and undo the damaged bonding. In the current investigation, the pushout bond strength value was much greater in the apical and middle thirds than in the coronal region.

The present study’s findings demonstrated that there was no discernible difference between Group 2 and Group 3 treated samples. This may be explained by the surface surfactant included in CHX composition, which raises dentin’s surface energy and, consequently, makes it more wettable, a quality necessary for resin sealer adherence. Additionally, CHX improved the dentin surface’s cationic charge and made the dentin substrate more favorable for bonding with AH Plus sealer, which can stick to the organic phase of radicular dentin [28,29,30].

The ability of a sealer to penetrate the dentinal tubules is desired because it would encapsulate remnant debris and micro-organisms and keep them apart from nutrition sources. Additionally, since it reduces the junction between gutta-percha and root dentin and may enhance the preservation of the filling mass by mechanical locking, extensive endodontic sealer penetration is crucial [31]. Only after smear layer is removed, the sealer’s capacity to enter tubular dentin and adapt the obturating component to the canal walls is significantly increased. AH Plus is a root canal sealant made of epoxy resin. High radio opacity, acceptable biocompatibility, low solubility, mild polymerization shrinkage, and micro-retention to root dentin are some of its other benefits. It also has strong mechanical qualities [32,33,34].

There have been studies that compared herbal irrigation’s pushout bond strength to that of mainstream endodontic irrigants. According to research by Al Azzawi et al., [35] the midroot dentin samples treated with herbal extracts (green tea and *Salvadora persica*) revealed dentin samples with a bond strength that was considerably higher than the waterlase group. However, Choudhury et al. [16] found that *Morinda citrifolia* juice (MCJ) and chitosan might boost sealer penetration and reduce the dislocation of obturating materials, even if EDTA was more effective in removing smear layers. Results of this investigation revealed that, when compared to MCJ and chitosan solution, EDTA had the highest pushout bond strength. Using *Azadirachta indica, Curcuma longa,* methyl ethylene diaminotetraacetic acid, and sodium hypochlorite (NaOCl) as the irrigating solution, Shweta et al. [36] evaluated the pushout bond strength of AH Plus sealant to dentin in a different investigation. *Azadirachta indica*, MTAD, and *Curcuma longa* were the next strongest pushout bond samples in the midroot region, followed by NaOCL samples. However, MTAD demonstrated the strongest binding strength in the cervical region, followed by *A. indica* and *C. longa.*

The use of a tooth model, even though better simulating clinical conditions, has some drawbacks. Results obtained in vivo could differ depending on a variety of possible factors, including the clinician’s irrigation preferences, the treatment protocol, the concentration of the endodontic irrigant, the tooth being treated, and canal morphology. The primary goal of the study was to determine whether different endodontic irrigant treatments on the internal dentinal surface had an impact on the characteristics of the endodontic sealers, even though the use of various final irrigation protocols in the assessed teeth would not necessarily replicate the clinical scenario. This might also be seen as a restriction in some ways as not all practitioners would adhere to the same irrigation schedules as those demonstrated in this study, which could vary among practitioners. The sealers utilized are another drawback; when used with other commercially available endodontic sealers, the outcomes simulated might not always be repeated. Further studies are required to determine if these characteristics are present with other commercially available sealers in various clinical circumstances, because this study was preliminary in nature and in vitro-based.

## 5. Conclusions

Under the constraints of this in vitro investigation, employing NaOCl as a final irrigant greatly reduces the dislocation resistance of AH Plus to root canal dentin. The impaired bond strength of AH Plus to NaOCl-treated dentin is also mitigated when *Arnica montana* and CHX are used as final irrigation solutions.

## Figures and Tables

**Table 1:** The mean, standard deviation, and statistical values of one-way ANOVA compared between the three groups (Group 1: sodium hypochlorite and saline; Group 2: *Arnica montana*; and Group 3: chlorhexidine).

Section	Coronal	Middle	Apical
Group 1	1.7630 ± 0.44502	5.2480 ± 0.65287	4.9960 ± 0.67145
Group 2	1.8730 ± 0.49376	4.4630 ± 0.85853	4.6010 ± 1.45383
Group 3	1.7850 ± 0.39697	4.3110 ± 0.91307	4.2390 ± 0.82174
*p* value (ANOVA)	0.845	0.035 *	0.282

* Statistically significant value in the middle section of Group 2.

**Table 2:** The multiple comparisons between the three groups (Group 1: sodium hypochlorite and saline; Group 2: *Arnica montana*; and Group 3: chlorhexidine).

Post Hoc Test (Paired Wise Comparison)	Group 1	Group 2	Group 3
Coronal vs. Middle	0.847	0.098	0.676
Coronal vs. Apical	0.993	0.041 *	0.251
Middle vs. Apical	0.899	0.909	0.719

* Statistically significant value in the apical section of Group 2.

**Table 3:** The modes of failure for the three groups (Group 1: sodium hypochlorite and saline; Group 2: *Arnica montana*; and Group 3: chlorhexidine).

Section	Group 1	Group 2	Group 3
Coronal	Adhesive 65	Adhesive 00	Adhesive 00
	Cohesive 10	Cohesive 40	Cohesive 35
	Mixed 20	Mixed 60	Mixed 55
Middle	Adhesive 55	Adhesive 20	Adhesive 10
	Cohesive 25	Cohesive 25	Cohesive 25
	Mixed 20	Mixed 70	Mixed 60
Apical	Adhesive 75	Adhesive 20	Adhesive 20
	Cohesive 15	Cohesive 35	Cohesive 55
	Mixed 20	Mixed 55	Mixed 55

## Data Availability

The data presented in this study are available on request from the corresponding author.

## References

[1] Verma D., Taneja S., Kumari M. (2018). Efficacy of different irrigation regimes on the push-out bond strength of various resin-based sealers at different root levels: An in vitro study. J. Conserv. Dent..

[2] Prado M., Gusman H., Gomes B.P.F.A., Simão R.A. (2011). Scanning electron microscopic investigation of the effectiveness of phosphoric acid in smear layer removal when compared with EDTA and citric acid. J. Endod..

[3] Shivanna V. (2014). The effect of different irrigating solutions on the push out bond strength of endodontic sealer to dentin and assessing the fracture modes: An in-vitro study. J. Int. Clin. Dent. Res. Organ..

[4] Haapasalo M., Shen Y., Wang Z., Gao Y. (2014). Irrigation in Endodontics. Br. Dent. J..

[5] Aboubakr R., Badaran A., Elghazawy R., El-Hady S. (2022). Evaluation of antibacterial effect against E. Feacalis and smear layer removal ability of turmeric extract solution as a root canal irrigant for primary anterior teeth: An in-vitro study. Adv. Dent. J..

[6] Melnyk N., Vlasova I., Skowrońska W., Bazylko A., Piwowarski J.P., Granica S. (2022). Current knowledge on interactions of plant materials traditionally used in skin diseases in Poland and Ukraine with human skin microbiota. Int. J. Mol. Sci..

[7] Mawardi H., Elbadawi L. (2022). A cross-sectional survey on dentist’s knowledge and attitude towards the application of Arnica Montana in dental practice. J. Clin. Diagn. Res..

[8] Koo H., Gomes B.P.F.A., Rosalen P.L., Ambrosano G.M.B., Park Y.K., Cury J.A. (2000). In vitro antimicrobial activity of propolis and Arnica Montana against oral pathogens. Arch. Oral Biol..

[9] Nabavizadeh M., Sobhnamayan F., Sedigh-Shams M., Liaghat S. (2022). Comparison of the push-out bond strength of ah plus sealer to dentin after using different herbal irrigation solutions as the final rinse. PLoS ONE.

[10] Collares F.M., Portella F.F., Rodrigues S.B., Celeste R.K., Leitune V.C., Samuel S.M. (2015). The influence of methodological variables on the push-out resistance to dislodgement of root filling materials: A meta-regression analysis. Int. Endod. J..

[11] Üreyen Kaya B., Keçeci A.D., Orhan H., Belli S. (2008). Micropush-out bond strengths of gutta-percha versus thermoplastic synthetic polymer-based systems—An ex vivo study. Int. Endod. J..

[12] Dem K., Wu Y., Kaminga A.C., Dai Z., Cao X., Zhu B. (2019). The push out bond strength of polydimethylsiloxane endodontic sealers to Dentin. BMC Oral. Health.

[13] Aamir M.N., Ahmad M., Ghafoor N. (2012). Antibacterial activity of mother tinctures of Cholistan desert plants in Pakistan. Indian J. Pharm. Sci..

[14] Fowler C.S., Swartz M.L., Moore B.K., Rhodes B.F. (1992). Influence of selected variables on adhesion testing. Dent. Mater..

[15] Yalgi V.S., Bhat K.G. (2019). Antibacterial activity of homoeopathic tinctures on bacterial strains of streptococcus mutans and enterococcus faecalis: An in vitro study. J. Clin. Diagn. Res..

[16] Shivanand S., Patil C., Patil A.C., Choudhury S., Patil S.A., Doddwad P.K. (2020). Evaluation of push-out bond strength of a resin sealer to dentin after a final flush of three irrigants. J. Contemp. Dent. Pract..

[17] Teixeira C.S., Alfredo E., Thomé L.H., Gariba-Silva R., Silva-Sousa Y.T., Sousa-Neto M.D. (2009). Adhesion of an endodontic sealer to dentin and gutta-percha: Shear and push-out bond strength measurements and Sem Analysis. J. Appl. Oral Sci..

[18] Gogos C., Economides N., Stavrianos C., Kolokouris I., Kokorikos I. (2004). Adhesion of a new methacrylate resin-based sealer to human dentin. J. Endod..

[19] Pawar A.M., Kfir A., Metzger Z., Bhardwaj A., Yohana Y., Wahjuningrun D.A., Luke A.M., Pawar B.A. (2022). Can type of instrumentation and activation of the final Irrigant improve the obturation quality in oval root canals? A push-out bond strength study. Biology.

[20] Nagas E., Cehreli Z.C., Durmaz V. (2011). Effect of light-emitting diode photopolymerization modes on the push-out bond strength of a methacrylate-based sealer. J. Endod..

[21] Pawar A.M., Pawar S., Kfir A., Pawar M., Kokate S. (2015). Push-out bond strength of root fillings made with c-point and BC sealer versus gutta-percha and AH plus after the instrumentation of oval canals with the self-adjusting file versus WaveOne. Int. Endod. J..

[22] Sritharan A. (2002). Discuss that the coronal seal is more important than the apical seal for endodontic success. Aust. Endod. J..

[23] Ørstavik D.A.G. (2005). Materials used for root canal obturation: Technical, biological and clinical testing. Endod. Top..

[24] Estrela C., Estrela C.R.A., Barbin E.L., Spanó J.C., Marchesan M.A., Pécora J.D. (2002). Mechanism of action of sodium hypochlorite. Braz. Dent. J..

[25] Daumer K.M., Khan A.U., Steinbeck M.J. (2000). Chlorination of pyridinium compounds: Possible role of hypochlorite, N-chloramines, and chlorine in the oxidation of pyridinoline cross-links of articular cartilage collagen type II during acute inflammation. J. Biol. Chem..

[26] Abuhaimed T.S., Abou Neel E.A. (2017). Sodium hypochlorite irrigation and its effect on bond strength to dentin. BioMed Res. Int..

[27] Kriplani P., Guarve K., Baghael U.S. (2017). *Arnica montana* L.—A plant of healing: Review. J. Pharm. Pharmacol..

[28] Tuncel B., Nagas E., Cehreli Z., Uyanik O., Vallittu P., Lassila L. (2015). Effect of endodontic chelating solutions on the bond strength of endodontic sealers. Braz. Oral Res..

[29] Bhandi S., Alkahtani A., Reda R., Mashyakhy M., Boreak N., Maganur P.C., Vishwanathaiah S., Mehta D., Vyas N., Patil V. (2021). Parathyroid Hormone Secretion and Receptor Expression Determine the Age-Related Degree of Osteogenic Differentiation in Dental Pulp Stem Cells. J. Pers. Med..

[30] Dinesh K., Murthy B.V.S., Narayana I.H., Hegde S., Madhu K.S., Nagaraja S. (2014). The effect of 2% chlorhexidine on the bond strength of two different obturating materials. J. Contemp. Dent. Pract..

[31] Ordinola-Zapata R., Bramante C.M., Graeff M.S., del Carpio Perochena A., Vivan R.R., Camargo E.J., Garcia R.B., Bernardineli N., Gutmann J.L., de Moraes I.G. (2009). Depth and percentage of penetration of endodontic sealers into dentinal tubules after root canal obturation using a lateral compaction technique: A confocal laser scanning microscopy study. Oral Surg. Oral Med. Oral Pathol. Oral Radiol. Endodontol..

[32] Andriukaitiene L., Song X., Yang N., Lassila L.V., Vallittu P.K., Kerosuo E. (2018). The effect of smear layer removal on E. faecalis leakage and bond strength of four resin-based root canal sealers. BMC Oral Health.

[33] Zanza A., Seracchiani M., Reda R., Miccoli G., Testarelli L., Di Nardo D. (2022). Metallurgical Tests in Endodontics: A Narrative Review. Bioengineering.

[34] Almadi K.H. (2022). Impact of antimicrobial photodynamic therapy on the bond-strength and penetration of endodontic sealers: A systematic review. Photodiagn. Photodyn. Ther..

[35] Al-Azzawi A.-K.J. (2014). The effect of waterlase laser and herbal alternative, green tea and Salvadora Persica (Siwak) extract on push-out bond strength. J. Baghdad Coll. Dent..

[36] Shweta C., Kimaya K.K., Chetana J., Alok R.P., Preetam P.S., Amol H.K. (2021). Comparison of the effect of different irrigating solutions on bond strength of obturating materials: An in vitro study. Int. J. Sci. Study.

